# Aeroacoustic source term computation based on radial basis functions

**DOI:** 10.1002/nme.6298

**Published:** 2020-01-20

**Authors:** Stefan Schoder, Klaus Roppert, Michael Weitz, Clemens Junger, Manfred Kaltenbacher

**Affiliations:** ^1^ Institute of Mechanics and Mechatronics TU Wien Vienna Austria

**Keywords:** compactly supported functions, computational aeroacoustics, multivariate interpolation, radial basis functions

## Abstract

In low Mach number aeroacoustics, the known disparity of length scales makes it possible to apply well‐suited simulation models using different meshes for flow and acoustics. The workflow of these hybrid methodologies include performing an unsteady flow simulation, computing the acoustic sources, and simulating the acoustic field. Therefore, hybrid methods seek for robust and flexible procedures, providing a conservative mesh to mesh interpolation of the sources while ensuring high computational efficiency. We propose a highly specialized radial basis function interpolation for the challenges during hybrid simulations. First, the computationally efficient local radial basis function interpolation in conjunction with a connectivity‐based neighbor search technique is presented. Second, we discuss the computation of spatial derivatives based on radial basis functions. These derivatives are computed in a local‐global approach, using a Gaussian kernel on local point stencils. Third, radial basis function interpolation and derivatives are used to compute complex aeroacoustic source terms. These ingredients are necessary to provide flexible source term calculations that robustly connect flow and acoustics. Finally, the capabilities of the presented approach are shown in a numerical experiment with a co‐rotating vortex pair.

## 1 INTRODUCTION

The demand for adapted interpolation techniques started with the need for topology interpolation. First attempts were made in terms of ordinary series representation. Trigonometric and polynomial series arise with an increasing computational effort and may not even represent typical surface elevations, such as hill tops and valleys. Regarding the needs, the application of radial basis functions began in the late 1960s in the field of geophysics. The appropriate treatment of scattered data is one of the main challenges when measuring air and sea temperature, wind speeds, or simply the topology of the earth. Having this scattered property of the data in mind, Hardy developed an approximation and interpolation method for surface fitting problems.^1^ Since then, the multiquadric approximation of scattered data has been used in geodesy, mapping, signal processing, digital terrain modeling, and hydrology.^2^ In the field of aeroelasticity, fluid‐structure interaction algorithms based on mesh‐less radial basis function (RBF) interpolation are proposed as a computational efficient coupling strategy.^3^, ^4^, ^5^ The coupling of nonconforming grids with focus on computational performance is of great importance in aeroelasticity.^6^

**Figure 1:**
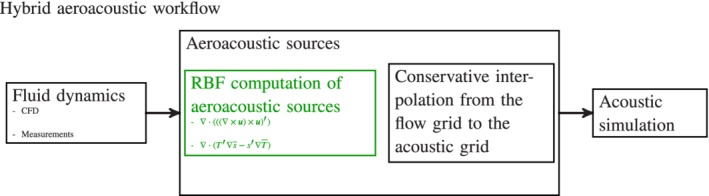
The hybrid aeroacoustic workflow consists of three main computational parts, where the presented radial basis function source‐term computation is a crucial part if the CFD solver provides only primary results, such as the pressure *p* or the velocity ***u***. Here, a prime denotes the fluctuating part of a physical quantity and a bar over a physical quantity its time average [Colour figure can be viewed at http://wileyonlinelibrary.com]

Today, efficient interpolation techniques are especially important when dealing with computational aeroacoustics. Since the beginning of CAA, hybrid methods have been established as the most practical for fast and accurate aeroacoustic computations at low Mach numbers. The workflow of hybrid aeroacoustics involves three steps (see Figure 1): (i) perform unsteady flow computations on a restricted subdomain; (ii) compute the acoustic sources; (iii) simulate the acoustic field. For low Mach number applications, a key challenge in computational aeroacoustics (CAA) is the huge disparity of scales between flow structures and audible acoustic wavelengths. The scaling between the acoustic wavelength λ and the characteristic length *l* of a vortex is given by

$$\lambda ∼\frac{l}{\text{Ma}},$$

where Ma is the Mach number. Utilizing this disparity of scales, aeroacoustic analogies or perturbation techniques separate the flow computation from the acoustic computation (eg, References 7, 8). Therefore, the sizes of the two computational grids are in general quite different.

For both physical fields, the individual optimal computational grid achieves the highest accuracy and the two grids differ according to the modeling criteria. On the one hand, the flow grid resolves boundary layers and is mostly coarsened toward outflow boundaries to dissipate vortices. On the other hand, the acoustic grid transports waves and therefore needs a uniform grid size all over the computational domain. In order to couple the different meshes, an interpolation scheme is necessary that satisfies the fundamental requirement of hybrid CAA: An accurate data transfer from the flow to the acoustic grid to minimize interpolation errors while conserving the energy. To cope with this task, different interpolation strategies can be applied, starting from low complexity nearest neighbor interpolation to complex source‐term computation models with volume intersections between flow and acoustic grid. The simple nearest neighbor interpolation fails to compute the acoustic sources accurately (eg, References 9, 10). In Reference 9, the aeroacoustic source term is computed by summing the contributions of all flow cells belonging to finite elements that surround an acoustic finite element node. It is assumed that the flow quantities used for the acoustic source‐term computation are constant over each flow cell. This approach has also been used in Reference 11 for three‐dimensional problems, where a grid dependency has been observed resulting in a too low sound pressure level over the whole frequency spectrum. A fully conservative approach has been used in Reference 12, where the acoustic sources within the finite element formulation are first computed on the fine flow grid. These so‐called nodal loads are then interpolated by a conservative scheme to the acoustic grid. This approach is accurate in cases where the flow grid is much finer than the acoustic grid but fails in cases where the flow grid is coarser than the acoustic grid. As a solution to this problem, we derived a cut cell approach and successfully applied it to the aeroacoustic computation of an axial fan.^13^ Similar investigations have been performed in Reference 10, where for both the flow and the acoustic field a finite volume scheme has been used. However, most of these methods fail if the computational fluid dynamics (CFD) simulation only provides primary variables, such as the pressure *p* or the velocity ***u***, since the aeroacoustic sources are often nontrivial combinations of these primary variables (see Figure 1).

Desirably, an accurate, flexible, and conservative coupling scheme ensures a rigorous connection between fluid dynamics and acoustics within a hybrid aeroacoustic simulation. The properties of the desired coupling scheme, or interpolation, are summed up as follows:

- An accurate and fast interpolation technique.
- An interpolation technique that handles special grids, for example, grids to resolve boundary layers.
- A method to compute accurate derivatives of the primary flow variables, such as pressure *p*, velocity ***u***, density ρ, temperature *T*, and entropy *s*.
- A flexible algorithm that can be integrated into a standard product development cycle.
- A flexible algorithm that assembles different hybrid aeroacoustic source terms, for example, the divergence of the Lamb vector ∇·(((∇×***u***)×***u***)^′^) or the divergence of an entropy source $\nabla \cdot (T^{\prime }\nabla \bar{s}-s^{\prime }\nabla \bar{T})$. 14
- A conservative algorithm that transfers the desired amount of energy, defined by the aeroacoustic sources, from the flow discretization to the mesh of the acoustic simulation (see eg, Reference 13;) is not discussed here.

In this paper, we propose to compute the aeroacoustic sources directly from the primary CFD variables by applying an interpolation scheme based on RBFs in conjunction with RBF derivatives. We show the ideal setup for the algorithms, the choice of the kernel function and propose a selection of discretization natural neighbors for the computation based on the connectivity. This patch search technique guarantees the resolution of typical flow structures. The application of local RBFs provides promising capabilities in terms of computational efficiency, known from nearest neighbor algorithms. Furthermore, the computation of RBF derivatives can be carried out elegantly and accurately with a local‐global approach.

The rest of the paper is organized as follows: In Section 2, we provide a general overview about the selected interpolation scheme, discuss convergence based on the parameters, the grid characteristics, the neighbor search, and typical fluid dynamic effects. Boundary layer interpolation motivates the proposed neighbor search technique and the superior performance is shown in a convergence study. Furthermore, the integration of constraints is discussed. Next, in Section 3, we discuss our approach to compute spatial derivatives based on local‐global RBFs, where we investigate the sensitivity of the scaling parameter based on verification benchmarks. The derivatives are judged by analytic representations of flow structures, such as a vortical distribution, a sinusoidal shock, and a boundary layer profile. Finally, in Section 5 we conclude the findings and give additional remarks for future research.

## 2 INTERPOLATION SCHEMES

**Figure 2:**
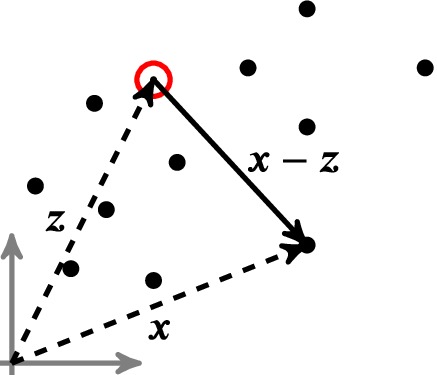
Geometric definitions of scattered data (***x***,•) and the target point (z,⊙). [Colour figure can be viewed at http://wileyonlinelibrary.com]

RBFs are scalar‐valued multivariant functions $R^{d}\mapsto R$ that depend on the radial measure *r* in terms of the Euclidean norm *r*=||***x***−***z***||_2_ between a scattered data (source) point ***x*** and the evaluation (target) point ***z*** (see Figure 2).

Over the years, application‐driven development of RBFs has modified function type, shape, and relevant support. The most prominent RBF kernels are the Gaussian, the multiquadric, and the inverse multiquadric.^15^

At this point, a clear distinction between local and global methods should be drawn. Global methods mainly using the Gaussian kernel are well suited for theoretical analyses since it can be shown that the solution converges to the exact solution under certain conditions.^16^ But on the downside, the condition number of the global interpolation matrix tends to infinity. There are some approaches to circumvent this issue^17^ using a QR‐decomposition or a Taylor‐expansion but they are computationally more expensive and less parallelizable than local ones.

With increasing number of ill‐placed datapoints in datasets, the demand for RBFs with compact local support^18^ increases. Local representations reduce computational time when interpolating data and increase the condition number of each local subproblem. Instead of inverting one ill‐conditioned global system matrix, many small well‐conditioned stencil‐matrices are inverted. Using Wendland's compactly supported RBFs together with a modified Shepard's method as presented in Reference 19, high computational efficiency for large system can be achieved.

An important aspect of this work is the application of RBF interpolation schemes to scattered data distributions with bad quality, such as extremely anisotropic point distribution, for example, from boundary layer flow. We use a local collocation method that represents the data exactly in the prescribed point. In this sense no algorithmic uncertainty is present at the individual, deterministic, scattered data point. To handle anisotropic point distributions, a special search procedure is used to find the optimal local point distribution for interpolation, which will be presented in Section 2.2.

### 2.1 RBF interpolation

As mentioned above, the main purpose of our method is to interpolate flow results, such as velocity ***u*** or vorticity **ω**, from a CFD mesh to an acoustic finite element mesh (FEM). Since hybrid aeroacoustics deals with a large number of unknowns and unfortunate data distributions, for example, in boundary layers, we focus on a parallelizable algorithm to achieve high computational efficiency.

In this work, the local Wendland kernel φ (1) together with a modified Shepard's method was chosen, similar to Reference 19. This is the most promising approach since it is both fast and capable of handling boundary layer meshes if the chosen kernel is combined with the patch search technique presented in Section 2.2.

The scaled Wendland kernel^19^ reads

(1) $$\varphi (||x-z|{|}_{2},\alpha )=\left(1-\frac{||x-z|{|}_{2}}{\alpha }\right),$$

where $x\in R^{d}$ is the location of a scattered data point. For all scattered data points the associated indices ω_*s*_∈1,2,…,*N*_*s*_ are defined, with *N*_*s*_ as the number of scattered data points, also called *source points*. The point $z\in R^{d}$ is the point at which the interpolation is evaluated. These points are called *target points* and form a set of target point indices ω_*t*_∈1,2,…,*N*_*t*_, with *N*_*t*_ as the number of target points. The parameter α is responsible for the scaling of the compact support (1), which is especially important for bad source point distributions resulting in a high condition number of the interpolation matrices. For our investigations, the parameter α is chosen to be

(2) $$\alpha ∼r_{\max}=\underset{i\in I_{q}}{\max}\{r_{i}\}\;,$$

with *I*_*q*_ as the indices of the *N*_*q*_ neighbors of the target point ***z*** and define the patch set. It should be noted that with this RBF interpolation, a higher order of accuracy can be achieved by “flattening” the compact support of the kernel (see Section 3.1) or increasing the order of the local interpolation kernel.

#### 2.1.1 Local interpolation method

Following the approach of Lazzaro,^19^ two different scattered data patches around the target point at which the interpolant is evaluated are introduced. The set *X*_*q*_={***x***_*i*_∈***x***,*i*∈*I*_*q*_}, with *I*_*q*_ as the indices of the *N*_*q*_ neighbors of the target point ***z***, defines the patch set. The set *X*_*w*_={***x***_*i*_∈***x***,*i*∈*I*_*w*_}, with *I*_*w*_ as the indices of the *N*_*w*_ influence points, defines the influence of the different patches. The influence radius $r_{W_{k}}=\max_{k\in I_{w}}\{r_{k}\}$, with *r*_*k*_=||***x***_*k*_−***z***||_2_, is defined to be the maximum distance of a target point in *X*_*w*_ and *N*_*w*_⊂*N*_*q*_. The interpolant is given by

(3) $$s(z)=\overset{N_{w}}{\underset{k=1}{\sum }}{\bar{W}}_{k}(z)R_{k}(z),$$

where *R*_*k*_(***z***) defines the local interpolation system and ${\bar{W}}_{k}$ the weight function. The local interpolation system is given by the algebraic system

(4) $$R_{k}(x_{k})=\overset{N_{q}}{\underset{j=1}{\sum }}c_{j}\varphi (||x_{j}-x_{k}|{|}_{2})\;,$$

which has to be initially solved for the *N*_*q*_ temporary (unscaled) interpolation weights *c*_*j*_, where *R*_*k*_(***x***_*k*_) denotes the scattered data (field that is interpolated) at the patch source point ***x***_*k*_. The weight function ${\bar{W}}_{k}$ of the modified interpolant *s*(***x***) is chosen to be

(5) $${\bar{W}}_{k}(x)=\frac{{\left(\max\left\{\frac{\left(r_{W_{k}}-r_{k}\right)}{r_{W_{k}}r_{k}},0\right\}\right)}^{p}}{\overset{N_{q}}{\underset{l=1}{\sum }}{\left(\max\left\{\frac{\left(r_{W_{k}}-r_{l}\right)}{r_{W_{k}}r_{l}},0\right\}\right)}^{p}}\;,$$

with the exponent *p* as a measure of locality. The bigger *p*, the more local the approach making it less accurate but capable of resolving stronger gradients. Since the weights ${\bar{W}}_{k}(x)$ constitute a partition of unity, the following holds:

(6) $$\overset{N_{w}}{\underset{k=1}{\sum }}{\bar{W}}_{k}(x)=1\;\text{.}$$

Then the interpolant *s*(***x***) can be evaluated at every target point ***z***. The algorithm of the local RBF interpolation is written in pseudo code (Algorithm 1).

Algorithm 1 **Local RBF Interpolation**

1

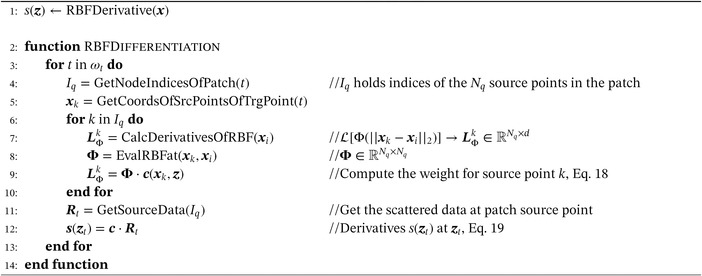

As described by Lazzaro in Reference 19, an upper bound for the global approximation error *E*(***x***) reads

(7) $$E(x)=\left|\left|f(x)-s(x)\right|\right|=\left|\left|\overset{N_{q}}{\underset{k=1}{\sum }}{\bar{W}}_{k}(x)f(x)-\overset{N_{q}}{\underset{k=1}{\sum }}{\bar{W}}_{k}(x)R_{k}(x)\right|\right|\leq \overset{N_{q}}{\underset{k=1}{\sum }}{\bar{W}}_{k}(x)e_{k}(x),$$

with $e_{k}(x)=\left|\left|f(x)-R_{k}(x)\right|\right|$ as the approximation error of the interpolant *R*_*k*_(***x***) relative to the local interpolation system. Additionally, Madych has proven in Reference 16 that the following holds if the distance *h* between the source points is sufficiently small:

(8) $$e_{k}(x)=\left|\left|f(x)-R_{k}(x)\right|\right|\leq C_{1}e^{-C_{2}\alpha }{\left|\left|f\right|\right|}_{2},$$

where $C_{1},C_{2}\in R^{+}$ are constants. Thus for the shape parameter of the Wendland kernel (1) α→∞, the approximation error of the local interpolation system *e*_*k*_(***x***) goes to zero and hence from Equation (7) it can be concluded that the same is true for the global approximation error *E*(***x***).

### 2.2 On the optimal choice of neighbors

An important part of the interpolation is the choice of source points ***x***_*i*_ in the patch *X*_*q*_ to avoid unphysical artifacts in the interpolated field. This is especially important in boundary layers, where cells or elements are distorted or stretched with high aspect ratios. A common approach to obtain a specific amount of nearest neighbors is to use a kd‐tree search, for example, from computational geometry algorithms library^20^ or fast library for approximate nearest neighbors. 21

**Figure 3:**
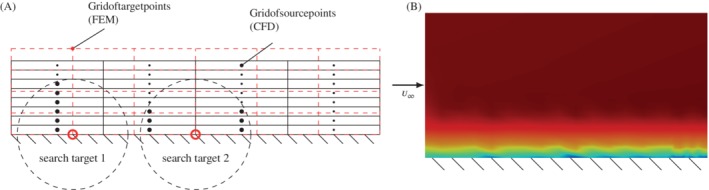
kd‐tree search and resulting interpolated flow field. A, The search of a kd‐tree patch is based on a metric (eg, euclidean) where all source points inside the influence domain (black dashed circle) surrounding the target point (red circle) are selected. For different mesh discretizations, different search patterns arise. These patterns severely influence the quality of the interpolation; B, The velocity field of a CFD simulation was interpolated to a different grid with the ordinary kd‐tree search algorithm. Nonphysical artifacts occur in the boundary layer

These fast kd‐tree algorithms have to be provided with the coordinate of the target point for which the patch is defined and all coordinates of the source points. By doing this, the underlying mesh structure (connectivity) is ignored and the data is represented on discrete scattered points (see Figure 3A). Thus, important information to improve the quality of the interpolation might be missed. To understand the shortcoming of this approach, a simple example of a boundary layer flow in a channel is provided, where the cell data from a CFD simulation is interpolated to nodes of a different FEM mesh. The interpolation from a CFD Finite Volume mesh to an FEM mesh is our standard procedure; however, the findings are also true for other discretization types without loss of generality.

The interesting part is the area close to the wall, where a mesh refinement is necessary to properly resolve the boundary layer. The resulting patch of elements emerging from a kd‐tree search for two example nodes on the boundary layer is shown in Figure 3A. There we can see the unfavorable choice of source points (black) for different target points (red circle). As shown in Figure 3B, this patch choice results in a poor interpolation in streamwise direction, which leads to numerical artifacts. The results become even worse if we provide a heavily distorted mesh. There we can observe that the patches only contain vertical neighbors and thus the patches have no connection in streamwise direction. A common approach to circumvent this issue is using anisotropic search algorithms and anisotropic kernel functions. In contrast to the herein described connectivity‐based neighbor search, additional tuning parameters have to be determined for local mesh features.

**Figure 4:**
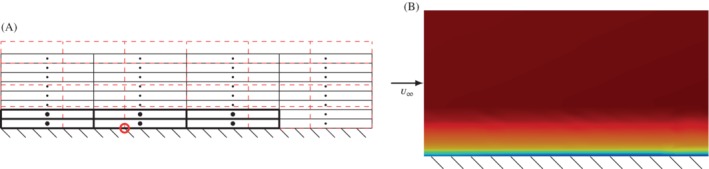
Modified patch search and resulting interpolated flow field. A, The modified patch search is based on the connectivity of the mesh. Only direct neighbors of the origin element are selected by a layer strategy. This ensures that the neighborhood discretizing the physical phenomena is used to interpolate the data and connections on meshes with high aspect ratios are maintained; B, The velocity field of a CFD simulation was interpolated to a different grid with the mesh based search algorithm. No artifacts occur in the boundary layer and the high quality of the flow field is maintained

However, if we use a connectivity preserving mesh‐based neighbor search, the patch looks as displayed in Figure 4A, which results in an even spatial distribution and good interpolation results (see Figure 4B). This modified patch algorithm searches for the next directly connected neighbor elements over the common global nodes. By applying a “layer strategy,” the neighbors of the direct neighbors are also taken into account. This search can be carried out recursively in a layered manner until the preferred number of elements is reached. In the illustrated example, only a one‐layer level search was applied.

Another drawback of a coordinate based kd‐tree search is the interpolation along slender wing profiles and structures. It is possible that a kd‐tree search at the upper side of the wing includes points from the lower side in the patch, which would be completely incorrect. In this case, the modified patch search based on the connectivity performs well since elements on the upper side have no direct connection to the elements on the lower side of the slender airfoil.

With the given interpolation formulation it is possible to impose exact boundary conditions, for example, a no‐penetration condition ***u***·***n***=0, where the corresponding entries of the interpolant can simply be set equal to zero.

### 2.3 Convergence of interpolation

To analyze the convergence of the RBF interpolation and to quantify the more or less heuristic observations of correct choice of patches, we prescribe analytic functions on a unit cube Ω∈[0,1]^3^ with different node distributions and observe the *L*_2_‐error after interpolating to a different mesh with equidistant node distribution.

**Figure 5:**
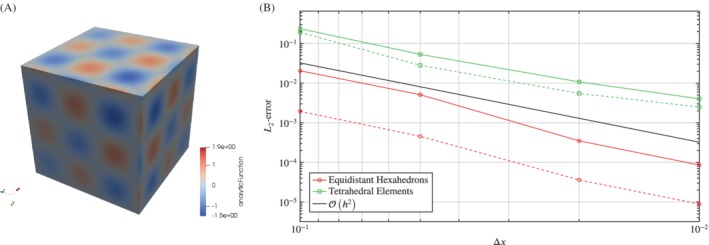
Convergence of the interpolation for the first analytic function $f_{1}\left(x\right)$. A, Representation of the first analytic function *f*_1_(*x*); B, Illustration of the *L*_2_‐error for the interpolation of the first analytic function on different mesh types using a nearest neighbor search (solid) and patch search (dotted). The target mesh discretization is Δ*z*=0.1

**Figure 6:**
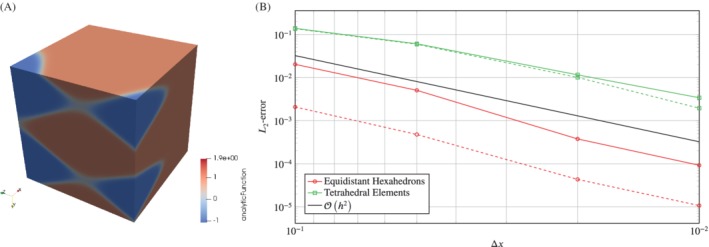
Convergence of the interpolation for the second analytic function $f_{2}\left(x\right)$. A, Representation of the second analytic function *f*_2_(*x*); B, Illustration of the *L*_2_‐error for the interpolation of the second analytic function on different mesh types using a nearest neighbor search (solid) and patch search (dotted). The target mesh discretization is Δ*z*=0.1

The first analytic function

(9) $$f_{1}\left(x\right)=\left(x^{2}+y^{2}+z^{2}\right)\;\sin\left(10\;x\right)\;\sin\left(10\;y\right)\;\sin\left(10\;z\right)$$

represents the transition of different vortex structures in shear layers (see Figure 5A). This smooth function is used to investigate the convergence and accuracy of the RBF interpolation since the majority of pressure fields encountered in low Mach number aeroacoustics are smooth. The second analytic function

(10) $$f_{2}\left(x\right)=\tanh\left(20\;\left(x+0.3\;\sin\left(-10\;y\right)-0.3\;\sin\left(-5\;\left(z-0.1\right)\right)\right)\right),$$

is a representative of a continuous sinusoidal shock, which is similar to the numerical solution of a shock using a dissipative flow solver^22^ (see Figure 6A). This function is used to test the capabilities of RBF interpolation to handle strong gradients and sharp transitions.

These analytic functions were prescribed on a unit cube Ω∈[0,1]^3^ consisting of equidistant hexahedrons, and tetrahedron elements. Then the analytic functions were interpolated to a different mesh consisting of equidistant hexahedrons with discretization distance Δ*z* using a nearest neighbor search or the modified patch search, respectively.

Figures 5B and  6B show the convergence of the RBF interpolation with respect to the source discretization distance Δ*x*, being the edge length of an element, for the first and second analytic function. Obviously, there is an influence resulting from the source data density, and the RBF interpolation performs well for both the nearest neighbor search and the modified patch search on all of the different mesh types. The larger *L*_2_‐error of the tetrahedron elements is due to an unsymmetric interpolation point distribution around the target point, which is suboptimal for RBF and leads to higher errors. However, the order of convergence remains the same.

In order to further analyze the capability of the RBF interpolation to handle boundary layer data in combination with the different choices of neighbors, we use the function

(11) $$f_{3}\left(x\right)=\frac{10}{4.6151}\left(\log(y+10^{-2})-\log(10^{-2})\right).$$

**Figure 7:**
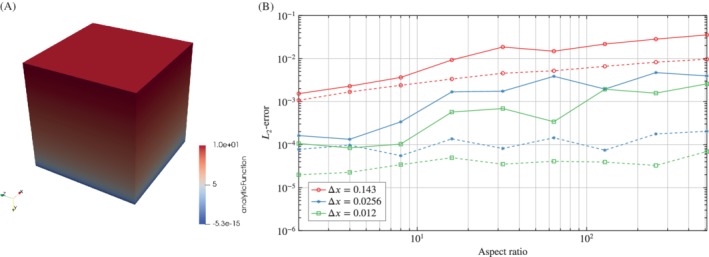
Convergence of the interpolation for the third analytic function $f_{3}\left(x\right)$ on a mesh to resolve a boundary layer. A, Representation of the third analytic function *f*_3_(*x*); B, Illustration of the *L*_2_‐error for the interpolation of the third analytic function on hexahedron mesh with different source discretizations using a nearest neighbor search (solid) and patch search (dotted). The target mesh discretization is Δ*z*=0.1

The analytic function $f_{3}\left(x\right)$ roughly describes the flow velocity component in inflow direction in a boundary layer (see Figure 7A). Here, the wall that is the reason for the boundary layer to develop is located at *y*=0. As mentioned above, a CFD mesh to properly resolve the boundary layer is characterized by cells with a big aspect ratio in the region adjacent to the wall where the largest gradients occur. For our investigations, we used a mesh consisting of regular hexahedrons with different aspect ratios in the range of 2 to 512 for the cells at *y*=0 (typical CFD boundary layer mesh^23^). With growing distance *y* the mesh gradually becomes coarser. In order to realize different aspect ratios in the first cell row, the cell height is varied while the number of nodes on the edges stays the same.

As shown in Figure 7B, the kd‐tree‐based nearest neighbor search interpolation causes increasing errors with increasing aspect ratio. Hence, the interpolation error for large aspect ratios limits the application of the nearest neighbor search. However, the previously described patch search leads to better results at large aspect ratios where it significantly outperforms the kd‐tree‐based nearest neighbor search. Ideally, the *L*_2_‐error is independent of the aspect ratio. One can see that this requirement is approximately met using patch search.

## 3 DERIVATIVES BASED ON RBFS

Although the procedure to compute derivatives based on RBFs is similar to the RBF interpolation, there is one important difference. For derivatives, there is only one source patch set *X*_*q*_={***x***_*i*_∈***x***,*i*∈*I*_*q*_}, with *I*_*q*_ as the indices of the *N*_*q*_ neighbors of the target point ***z***.

To explain the derivative of a given function *s*(***z***) at target point ***z***, let us start using matrix notation. We first need to interpolate the values at discrete datapoints ***x*** to determine the interpolation weights **σ**. Thus we need to form the interpolant ***s***(***x***)

(12) s(x)=Φ(x)·σ,

and solving this system for the weights **σ** leads to

(13) $$\sigma =\Phi^{-1}(x)\cdot s\;.$$

Since we want to apply the differential operator ℒ[] to the interpolant, we may write

(14) ℒ[s(z)]=ℒ[Φ(z)·σ],

and inserting the weights from Equation (13) yields

(15) $$ℒ[s(z)]=ℒ[\Phi (z)\cdot \Phi^{-1}(x)\cdot s].$$

Now we can use the fact that **σ**=**φ** ^−1^(***x***)·***s*** does not depend on ***z*** and we can pull it out of the operator's range

(16) $$ℒ[s(z)]=ℒ[\varphi (z)]\cdot \underset{\sigma }{\underbrace{\varphi^{-1}(x)\cdot s}},$$

and we can rewrite more compactly in the following way:

(17) $$ℒ[s(z)]=\underset{c(z)}{\underbrace{ℒ[\varphi (z)]\cdot \varphi^{-1}(x)}}\cdot s\;.$$

The process from above can now be transformed to a more implementation‐friendly form, based on Reference 24, where we first have to apply the spatial derivative operator ℒ to the RBF and evaluate it, as presented in Equation (18), where ***x***_*k*_ and ***x***_*l*_ are the coordinates of two source points with indices *k*,*l*∈*I*_*q*_.

(18) $$L_{\varphi }^{k}=ℒ[\varphi (||z-x_{k}|{|}_{2})]=\overset{N_{q}}{\underset{l=1}{\sum }}c_{l}\varphi (||x_{l}-x_{k}|{|}_{2})\;,\;k=1,\ldots ,N_{q}\;,$$

whereas the derivative of the Gaussian kernel was computed analytically. This system has to be inverted in order to obtain the weights *c*_*l*_ for the derivatives at the source points *l*∈*I*_*q*_. Then the derivative of the given function *s*(***z***) can be evaluated by

(19) $$ℒ[s(z)]=\overset{N_{q}}{\underset{l=1}{\sum }}c_{l}s(x_{l})=\overset{N_{q}}{\underset{l=1}{\sum }}c_{l}R_{l}\;,$$

where $R_{l}=s(x_{l})\in R^{N_{q}\times 1}$ is the scattered data value of the given function at the source point with index *l*.

Algorithm 2 shows how to calculate derivatives of scattered data distributions. Vector‐valued datasets can be interpreted as a tuple of scalar components

(20) $$s(x)=\left(\begin{array}{c} s_{1}(x)\\ s_{2}(x)\\ s_{3}(x) \end{array}\right)\;.$$

Then ***R***_*k*_ is a matrix $R^{N_{q}\times d}$ with *d* as the spatial dimension and evaluating the last expression of Algorithm 2 leads to a Jacobian matrix (21), where each row *i* contains the derivatives of the scattered data vector.

Algorithm 2 **Local RBF Derivative**

1

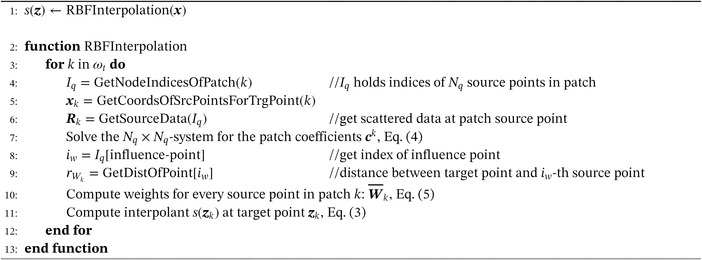

To show how to form the desired differential operator from the spatial differentiation matrix and to outline the workflow, a simple example is presented. Assume we have a scattered data vector field ${\left(s_{1},s_{2},s_{3}\right)}^{T}(z)\in R^{3}$. Then the rows of $L_{\varphi }^{i}$ contain the spatial derivatives of the three‐dimensional RBF in the three spatial directions $\left(\frac{\partial \varphi }{\partial z_{1}},\frac{\partial \varphi }{\partial z_{2}},\frac{\partial \varphi }{\partial z_{3}}\right)$. Additionally, the derivative coefficients ***c***[*i*] for source point *i* are three‐dimensional vectors. The final evaluation ***S***(***z***)=***c***·***R***(***z***) leads to the following 3×3 matrix

(21) $$\left(\begin{array}{ccc} \frac{\partial s_{1}}{\partial z_{1}} & \frac{\partial s_{1}}{\partial z_{2}} & \frac{\partial s_{1}}{\partial z_{3}}\\ \frac{\partial s_{2}}{\partial z_{1}} & \frac{\partial s_{2}}{\partial z_{2}} & \frac{\partial s_{2}}{\partial z_{3}}\\ \frac{\partial s_{3}}{\partial z_{1}} & \frac{\partial s_{3}}{\partial z_{2}} & \frac{\partial s_{3}}{\partial z_{3}} \end{array}\right).$$

### 3.1 Kernel scaling

As mentioned above, we use a Gaussian kernel in a local approach. The local kernel has no numerical stability problem in its basic form and works well on regular grids. But as soon as the mesh becomes distorted (eg, boundary layer) the results worsen. An improvement can be achieved by scaling the Gaussian ansatz function in the following way:

(22) $$\varphi \left(||x-z|{|}_{2},\alpha \right)=e^{{\left(\frac{||x-z|{|}_{2}}{\alpha }\right)}^{2}}\;.$$

We found that the Gaussian kernel theoretically leads to high accuracy (in theory we can differentiate up to any given order) but the stability problem, similar to the global interpolation method, restricts the maximum flatness α. Roberts proposed a method using a Taylor's expansion in the flatness parameter α to circumvent this issue.^25^ However, there are optimization strategies^15^ to find optimal values of the flatness parameter α.

It should be emphasized here that the parameter α is computed differently depending on whether an interpolation or derivation procedure is carried out. Our idea to find an optimal shape parameter for the RBF derivative procedure is to derive a relationship (a confident initial guess)

(23) $$\alpha ∼f(r_{12}),$$

for the parameter α with respect to the value *r*_12_ as the smallest distance between two points in the stencil of the current target point. The basic approach is to minimize the following objective functional

(24) $$E(\alpha ,r_{12})+\lambda (\kappa (\alpha ,r_{12})-\kappa_{\text{limit}})\;,$$

with the *L*_2_‐error *E*, the Lagrange multiplier λ, the condition number κ, and the limit of the condition number κ_limit_ where the system becomes sensitive to floating‐point round‐off errors. The *L*_2_‐error represents the deviation of the basis function from being one (absolutely flat, α→∞) on the whole domain. This error measure can simply be obtained by integrating

(25) $$E(\alpha ,r_{12})=\int_{\Omega_{2/3}}|1-\varphi (||x-z|{|}_{2},\alpha ){|}^{2}d\Omega \;.$$

The challenging task is to find the influence of the shape parameter α on the condition number of the resulting linear system. In general, the condition number κ of a matrix is defined as the ratio of the largest to the smallest eigenvalue:

(26) $$\kappa (\alpha ,r_{12})=\frac{\sigma_{\max}(\alpha ,r_{12})}{\sigma_{\min}(\alpha ,r_{12})}\;.$$

Therefore, the smallest eigenvalue must be nonzero in order to stay invertible.

The minimization is trivial since the functional decreases monotonically in α while a critical condition number κ_limit_ guarantees the invertibility of the matrix. With this constraint, our confident initial guess α_initial_ is

(27) $$\alpha_{\text{initial}}∼1/r_{12}$$

and from this starting point the shape parameter is iterated to the limit of the matrix invertibility, depending on the inversion algorithm.

As proven multiple times in the literature dealing with Gaussian RBF kernels (eg, in Reference 19), the *L*_2_‐error decreases with increasing shape parameter α. However, this is true only up to a certain value, where numerical instabilities tend to increase the error again.

### 3.2 Additional notes

During the computation, there is an easy way to assess how accurate the derivative will be. Looking at the last expression of Algorithm 2, where we multiply the weight matrix $c\in R^{d\times N_{q}}$ and the source data matrix $R\in R^{N_{q}\times d}$, we can state that the rowsum of each row must be zero. This can be seen easily by considering a constant field, where all entries are equal *R*_*ij*_=const. and the resulting derivative must be zero (derivative of a constant function) $S_{i}(z)=\sum_{j=0}^{N_{q}}c_{ij}R_{j}=0$. For nontrivial coefficients, this only holds if the rowsum is zero $\sum_{j=0}^{N_{q}}c_{ij}=0$ for *i*=1,…,*d*. This rowsum condition is used for optimal neighbor consideration.

During the investigations, the local Wendland kernel $\varphi_{2,0}={\left(1-||x-z|{|}_{2}\right)}^{2}$ works perfectly for generic test cases with moderately distorted elements. But as soon as the Wendland kernel is applied to more sophisticated problems with boundary layers, severe numerical artifacts are observed, especially when computing the divergence. Therefore a local approach with a Gaussian kernel (22) is used, which produces accurate results for a wide range of mesh discrepancies.

### 3.3 Convergence of derivatives

To analyze the convergence and accuracy of the RBF derivatives, we again prescribe our analytic functions Equations (9) to (11) on a unit cube Ω∈[0,1]^3^ with different node distributions using the patch search algorithm and observe the *L*_2_‐error after computing the gradient of the field.

**Figure 8:**
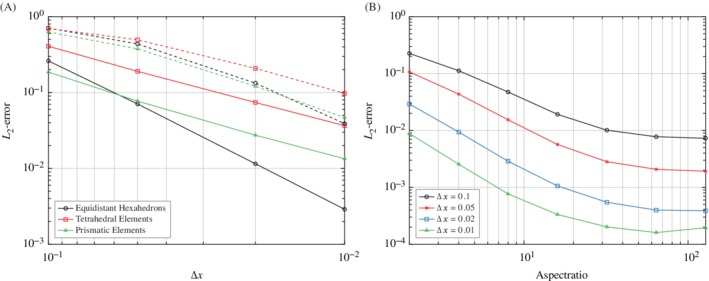
Convergence of the derivatives for the analytic functions *f*_1_ (9), *f*_2_ (10) and *f*_3_ (11). A, Illustration of the *L*_2_‐error for the gradient of the first analytic function *f*_1_ (solid) and the second analytic function *f*_2_ (dashed) with respect to the mesh discretization; B, Illustration of the *L*_2_‐error for the gradient of the third analytic function *f*_3_ on a hexahedron mesh with respect to the mesh discretization [Colour figure can be viewed at http://wileyonlinelibrary.com]

Figure 8A shows the convergence of the RBF derivatives with respect to the characteristic discretization distance Δ*x* for the first and second analytic function. Similar to the RBF interpolation, there is an influence resulting from the data density. Although the RBF derivatives are accurate for both analytic functions, it is obvious that larger gradients (as encountered in the second analytic function) lead to larger deviations.

Additionally, the convergence of the derivatives in boundary layers was analyzed considering the analytic function (11). This analytic function was again prescribed on a mesh consisting of regular hexahedrons with different aspect ratios in the range of 2 to 128 for the cells in the first row.

As shown in Figure 8B, the *L*_2_‐error decreases with increasing number of nodes in the region where the largest gradients occur (next to the wall). However, since the number of nodes on the edges perpendicular to the wall stays the same while the height of the cells is varied, a point is reached eventually where the nodes are not distributed well enough over the entire region of the largest gradients. This is the reason for the flattening of the curves for hexahedrons with large aspect ratios.

## 4 NUMERICAL EXAMPLE

The rotating vortex pair has been frequently used to determine the capabilities of aeroacoustic methodologies.^26^, ^27^, ^28^, ^29^ This arrangement has the nature of a quadrupolar sound field.

**Figure 9:**
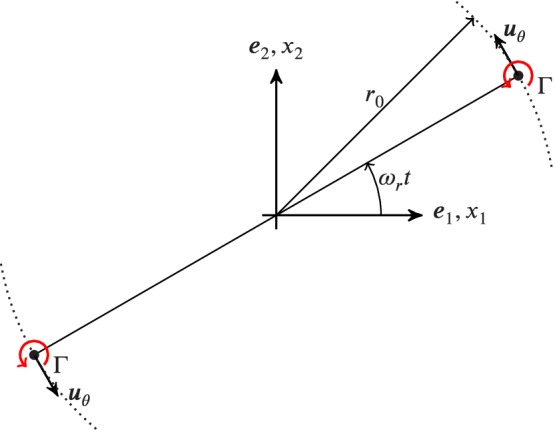
Schematic of the co‐rotating vortex pair defining the main geometrical and physical characteristics [Colour figure can be viewed at http://wileyonlinelibrary.com]

Figure 9 illustrates the configuration of the vortex pairs. Both vortices are delta distributions and oppose each other at a distance of 2*r*_0_. The strength of each vortex is characterized by the circulation intensity Γ. The vortices rotate around the origin with a period of $T=8\pi^{2}r_{0}^{2}/\Gamma$ imposing an angular rotating speed $\omega_{r}=\Gamma /(4\pi r_{0}^{2})\cdot e_{3}=\omega_{r}\cdot e_{3}$. Each vortex convects the other vortex by a velocity of ***u***_θ_=Γ/(4*πr*_0_)·***e***_*t*_, where ***e***_*t*_ is the unit vector in tangential direction. The Mach number in the circumferential direction is given by *M*_θ_=*u*_θ_/*c*= Γ/(4*πr*_0_ *c*). As already mentioned, the hydrodynamic field of the spinning vortex pair is expressed by a rotating quadrupole field. The potential flow theory can be used to determine the fundamental solution of the spinning vortex pair in terms of the complex flow potential function. In doing so, we introduce the transformation from Cartesian coordinates (*x*_1_,*x*_2_) to the complex plane with the complex coordinate $z=r\exp^{i\theta }=x_{1}+ix_{2}$. The location of each vortex over time *t* is defined by $b=r_{0}\exp^{i\omega t}$. Using the definitions, we express the incompressible, inviscid flow potential *ϕ*(*z*,*t*) as

(28) $$\varphi (z,t)=\frac{\Gamma }{2\pi i}\ln(z-b)+\frac{\Gamma }{2\pi i}\ln(z+b)=\frac{\Gamma }{2\pi i}\ln(z^{2}-b^{2})\;.$$

From this field we can derive the primary variables representing the fluid dynamic quantities. Based on the pressure and the velocity, aeroacoustic sources are derived. The incompressible velocity field **u**=(*u*_1_,*u*_2_)^T^ of the spinning vortex pair is obtained by differentiating Equation (28) with respect to the complex coordinate *z*

(29) $$u_{1}-iu_{2}=\frac{\partial \phi (z,t)}{\partial z}=\frac{\Gamma }{\pi i}\frac{z}{z^{2}-b^{2}}\;.$$

The fluid dynamic pressure *p* ^ic^ is obtained by applying the unsteady form of Bernoulli's principle

(30) pic=p0−ρ0∂Re{ϕ(z,t)}∂t−12ρ0(u12+u22).

Müller and Obermeier^30^ derived an analytic solution of the acoustic far‐field, based on matched asymptotic expansion of the potential solution. Starting from the solution in form of a complex potential (28), matching the inner and the outer solution yields the far‐field of the acoustic pressure fluctuation *p′* of the co‐rotating vortex pair

(31) $$p^{\prime }=\frac{\rho_{0}\Gamma^{4}}{64\pi^{3}r_{0}^{4}c^{2}}\left(J_{2}(2kr)\cos(2(\omega t))-Y_{2}(2kr)\sin(2(\omega t))\right)\;.$$

In Equation (31) *k*=ω/*c* denotes the wave number, *J*_2_(⋆) the second‐order Bessel function of first kind and *Y*_2_(⋆) the second kind. This analytic pressure fluctuation *p′* is used to verify both aeroacoustic methodologies, that is, using the divergence of Lamb vector and the first substantial time derivative of the incompressible pressure as acoustic source terms. It should be emphasized that this fluctuating pressure *p′* is not equal to the acoustic pressure *p* ^a^; however, *p′*→*p* ^a^ holds in the far‐field.

### 4.1 Numerical investigation

In order to judge the computational procedure,^26^, ^27^, ^28^, ^29^ we compare the analytic solution of the test case with a numerically obtained solution based on the simulation procedure that is illustrated in Figure 1. This analytic validation has the benefit of mitigating experimentally induced errors, reducing the overall modeling uncertainty of the aeroacoustic model, and simply avoiding errors of a numerical benchmark routine. We simulated the co‐rotating vortex pair on a stationary grid with moving sources induced by the vortical structures. An unstructured mesh is used to discretize the computational domain. In the source region, a characteristic mesh size of *h*≈90 cm is used. Each vortex distribution Γδ(*z*−*b*) is approximated by a continuous multivariant normal distribution with equivalent circulation Γ and an isotropic variance of σ^2^=0.05 m^2^.

We assume that the analytic field (***u***, *p* ^ic^) is represented on the flow grid and is based on a circulation strength of Γ=2π m^2^/s and a distance of 2*r*_0_=2 m between the vortices. The angular rotation induced by the vortices is ω_*r*_=0.5/s, the speed of sound $c=\sqrt{10}\;\text{m/s}$ and density $\rho_{0}=1\;{\text{kg/m}}^{3}$. Using this flow field, we apply the source term computation procedure and simulate the sound which is compared to the analytic solution in the far‐field (31). We verify the interpolation methodology by two hybrid aeroacoustic simulations of the co‐rotating vortex pair. The first simulation is based on vortex sound and the divergence of the Lamb vector as source term^31^

(32) $$\frac{1}{c^{2}}\frac{\partial^{2}p^{\prime }}{\partial t^{2}}-\Delta p^{\prime }=\rho_{0}\nabla \cdot (\omega \times u)\;.$$

The second verification example is based on the perturbed convective wave equation (PCWE) with the substantial derivative of the incompressible pressure as aeroacoustic excitation^13^

(33) $$\frac{1}{c^{2}}\frac{D^{2}\psi^{a}}{Dt^{2}}-\Delta \psi^{a}=\frac{1}{\rho_{0}c^{2}}\frac{Dp^{\text{ic}}}{Dt}\;.$$

In Equation (33), ψ^a^ denotes the acoustic velocity potential and $\frac{D}{Dt}=\frac{\partial }{\partial t}+\bar{u}\cdot \nabla$ the substantial derivative. The acoustic pressure can be computed as $p^{a}=\rho_{0}\frac{D\psi^{a}}{Dt}$.

Based on the computational methodology of RBFs, we propose the following simulation workflow for the computation of the aeroacoustic source terms. First, the primary fields incompressible pressure *p* ^ic^, flow velocity ***u*** ^ic^, and vorticity **ω** are computed in a flow simulation on the CFD mesh. Second, if required, derivatives are computed by the RBF framework and the source term is assembled on the CFD mesh. Naturally, the RBF framework is carried out on the discretization of the flow simulation, since this discretization already resolves characteristic flow structures. As a third step, a conservative integration^13^ of the source terms ensures a nondissipative calculation of the aeroacoustic finite element sources that are applied to the finite element simulation. Finally, the acoustic simulation is carried out, typically on a much coarser CAA mesh.

The source term of Equation (32) involves the cross‐product to determine the Lamb vector **ω**×***u*** and employing RBF derivatives yields the divergence of the Lamb vector ∇·(**ω**×***u***). Of course, the presented framework is capable of calculating the vorticity as the curl of the velocity field **ω**=∇×***u***, with the source term ∇·((∇×***u***)×***u***).

For the PCWE (33), the proposed workflow is as follows. The substantial derivative of the incompressible pressure is computed from primary CFD quantities. With the use of RBF derivatives, the gradient of the incompressible pressure is evaluated and the inner product with the velocity field is computed. We utilize a fifth‐order backward differencing scheme for the time derivative of the pressure. Then the source term is assembled for the acoustic simulation.

For both acoustic simulations, a Newmark scheme with a time step size of Δ*t*=0.09 s is applied for the time discretization. The computational domain (260x260m) is structured in three separate conforming subdomains. A source domain consisting of a disk with a diameter of 5*r*_0_ (relatively high resolved unstructured triangular mesh, *h*≈90 cm) is embedded in a propagation region, in which we gradually increase the unstructured mesh size. The propagation region is surrounded by a structured perfectly matched layer region,^32^ which absorbs the radiating waves. The wave length $\lambda =\frac{2\pi }{k}\approx 20\;m$ is resolved (in the propagation region) with approximately 20 linear finite elements per wavelength.

**Figure 10:**
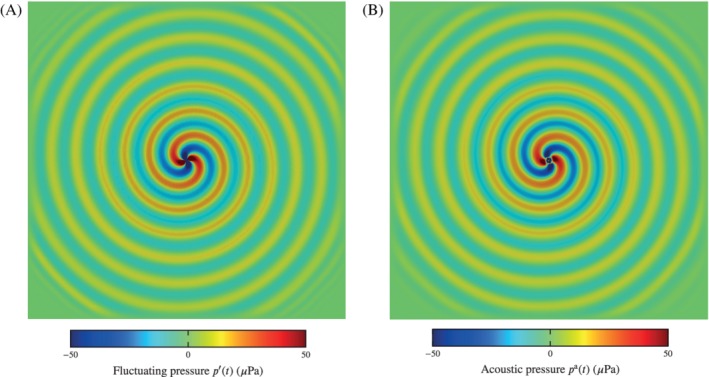
Acoustic fields of the co‐rotating vortex pair. A, Result of Equation 32 with the Lamb vector as source term; B, Result of Equation 33 with the substantial derivative of the incompressible pressure as source term

Figure 10A shows the acoustic field of co‐rotating vortex pair, with the Lamb vector as source term. The acoustic field with the substantial derivative as source term is depicted in Figure 10B. In the acoustic near‐field, differences occur due to the different solution quantities in the two aeroacoustic formulations (fluctuating pressure *p′* with the divergence of the Lamb vector as source term and acoustic pressure *p* ^a^ for the PCWE). Both results have the characteristic radiation pattern of the co‐rotating vortex pair.

**Figure 11:**
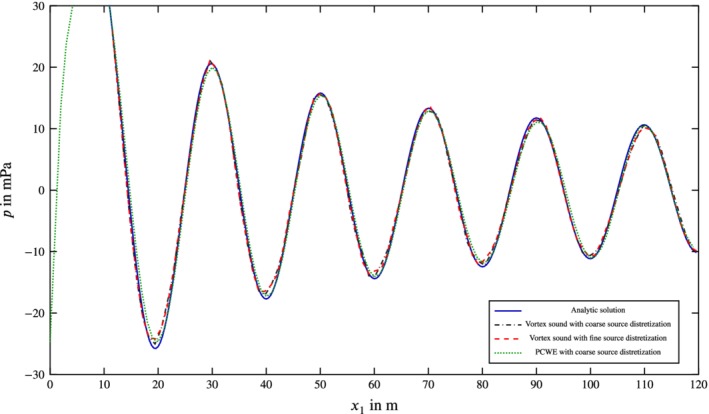
Fluctuating pressure *p* ^*′*^ or acoustic pressure *p* ^a^, respectively, as a function of the coordinate *x*_1_. Both the coarse and the fine discretization are simulated with the divergence of the Lamb vector as right‐hand side of the wave equation and compared to the sound field of the perturbed convective wave equation as well as the analytic solution [Colour figure can be viewed at http://wileyonlinelibrary.com]

The steepest descent of the Gaussian distribution is discretized by five linear triangular elements over 2σ. This coarse approximation of the vortical distribution shows the robustness of the RBF derivatives. A mesh refinement of the source region (see Figure 11) causes no significant increase in accuracy compared to the analytic solution. As depicted in Figure 11, one can clearly see the good accordance of numerical and analytic solution, even for the coarse grid.

## 5 SUMMARY AND CONCLUSION

In this work, we have presented how to use RBFs in a local formulation for the interpolation of scattered data using a Wendland kernel in combination with a modified Shepard's method. Since hybrid aeroacoustics deals with a large number of unknowns and unfortunate data distributions, for example, in boundary layers, we focused on a parallelizable algorithm to achieve high computational efficiency in regards of algorithmic complexity. For the presented approach, an algorithmic complexity of $𝒪(N\cdot N_{q})$ can be estimated, where *N* is the number of interpolation points and *N*_*q*_ the size of the local stencils. Since for large datasets *N*≫*N*_*q*_, the scaling of the presented method behaves better in comparison to classical (global) RBF interpolation, which has complexity $𝒪(N^{2})$.

The choice of the local interpolation patches is very important for the interpolation of distorted meshes, as they occur for example in boundary layers of flow simulations. Therefore, we proposed a connectivity preserving, mesh‐based neighbor search. This modified patch algorithm searches for the next directly connected neighbor elements over the common global nodes. Additionally, the neighbors of the direct neighbors are also taken into account by applying a *layer strategy*. To analyze the convergence of the RBF interpolation and to quantify the choice of neighbors, analytic functions were prescribed on a unit cube with different node distributions and the *L*_2_‐error was observed after interpolating to a different mesh with equidistant node distribution. In doing so, we found that both search methods perform well for well distributed data points. However, for large aspect ratios of boundary layer meshes, the connectivity preserving, mesh‐based neighbor search significantly outperformed the kd‐tree based nearest neighbor search. Furthermore, constraints and boundary conditions are incorporated to enhance the interpolation of boundary layers.

Additionally to RBF interpolation, we have presented how to compute spatial derivatives based on RBFs. The derivatives were computed in a local‐global approach, using a Gaussian kernel on local point stencils. Investigations on the local‐local approach, using a Wendland kernel on a local point stencil, showed reduced accuracy for skewed grids. Moreover, an optimization strategy to find an optimal value of the shape parameter scaling the Gaussian kernel was presented. Good convergence of this scaled exponential kernel was shown for the computation of the gradient of an analytic field that was prescribed on a unit cube with different node distributions.

Finally, a numerical investigation with a co‐rotating vortex pair was carried out to show the proposed workflow from CFD results to aeroacoustics using RBFs. This example also included the comparison to an analytic solution, which showed excellent agreement even for relatively coarse aeroacoustic meshes.
